# A novel approach of manual manipulation of the transnasal ileus tube for severe or recurrent benign adhesive small bowel obstruction

**DOI:** 10.3389/fsurg.2025.1601111

**Published:** 2025-09-15

**Authors:** Er-Sheng Li, Yin-Jun Zhai, Yin Han, Qian Chang, Qi Wang, Hong-Yu Zhang

**Affiliations:** Department of Radiology, The Second Affiliated Hospital, Xingtai Medical College/Xingtai Cancer Hospital, Xingtai, Hebei Province, China

**Keywords:** small bowel obstruction, ileus tube, adhesion, manual manipulation, draw back

## Abstract

**Purpose:**

This study seeks to evaluate the safety and efficacy of an innovative manual manipulation technique for the transnasal ileus tube in patients experiencing severe or recurrent adhesive small bowel obstruction (ASBO).

**Materials and Methods:**

Prior to the initiation of this research, approval was obtained from the institutional review board. The study was conducted within our institution, involving a cohort of fifty-four patients diagnosed with severe, multiple, or recurrent ASBO. These patients underwent treatment through active manipulation of the transnasal ileus tube, which entailed navigating obstructions by adjusting two balloons and resolving adhesions through the alternate advancement and retraction of the ileus tube. Angiographic outcomes were categorized as either complete or incomplete resolution of obstructions. Data were collected on technical success, initial and final angiographic outcomes, mortality, morbidity, and overall clinical outcomes. Follow-up assessments were conducted at 1, 3, 6, and 12 months, with annual evaluations thereafter.

**Results:**

The transnasal ileus tube was successfully placed in all patients without any procedure-related complications. The tube was successfully navigated and passed through obstructions to the cecum in 94.4% of cases, specifically in 51 out of 54 attempts. Follow-up angiograms, conducted over a period of 3–38 months, confirmed the unobstructed passage of contrast medium through the small bowel in 52 patients. Clinical follow-up data, with an average duration of 20 ± 11 months (95% CI: 17, 23 months; range, 6–45 months), were available for 52 patients. One patient died due to multiple organ failure, while the remaining 51 patients exhibited no clinical symptoms of small bowel obstruction.

**Conclusion:**

These preliminary findings indicate that manual manipulation of the transnasal ileus tube is non-surgical and therefore non-traumatic but nonetheless an effective method of internally lysing the adhesions with their double balloon technique and pulling the tube back and forth.

## Introduction

Small bowel obstruction (SBO) is the most frequently encountered surgical complication involving the small intestine (1–3). Intra-abdominal adhesions, which often result from previous abdominal surgeries, account for up to two-thirds of SBO cases, leading to what is known as adhesive small bowel obstruction (ASBO) (3–5). ASBO is an uncomfortable diagnosis and treatment involves NG tube which can be prolonged and ineffective, not resolving the underlying adhesions; or it needs surgery which is more definitive, but has complications and can cause more scar tissue (6–8).

Since the 1930s, favorable outcomes have been acquired in the management of ASBO through a long ileus tube to decompress the obstructed intestine (9–14), however, there remains a subset exceeding 10% of ASBO patients who do not benefit from long nasointestinal tubes and consequently require surgical intervention. To address these challenges, we devised a pioneering technique involving the manual manipulation of an ileus tube to navigate through the obstruction and alleviate the ASBO. This study aims to introduce these novel techniques and assess their safety and efficacy in a cohort of 54 patients with severe or recurrent ASBO.

## Materials and methods

### Patients

The study protocol was approved by our institutional review board, and written informed consent was obtained from all eligible participants prior to their inclusion in the study. Between April 2017 and December 2023, a total of 54 patients diagnosed with severe, multiple, or recurrent adhesive small bowel obstruction (ASBO) were enrolled and subsequently monitored at our institution.

The inclusion criteria were as follows: 1. prior surgical history; 2. the presence of definitive clinical symptoms and physical signs indicative of mechanical intestinal obstruction, such as abdominal pain and distension, nausea, vomiting, and constipation; 3. a diagnosis of benign adhesive ASBO confirmed through abdominal plain radiographs and computed tomography (CT) scans, corroborated by at least two attending radiologists; and 4. the availability of at least one control angiogram conducted a minimum of three months post-treatment. Patients with suspected postoperative peritoneal cavity infection, paralytic ileus, strangulated intestinal obstruction, adynamic obstruction, or ileus due to volvulus, recurrent tumor, or hernia were excluded from the study.

### Ileus tube

**Figure 1:**
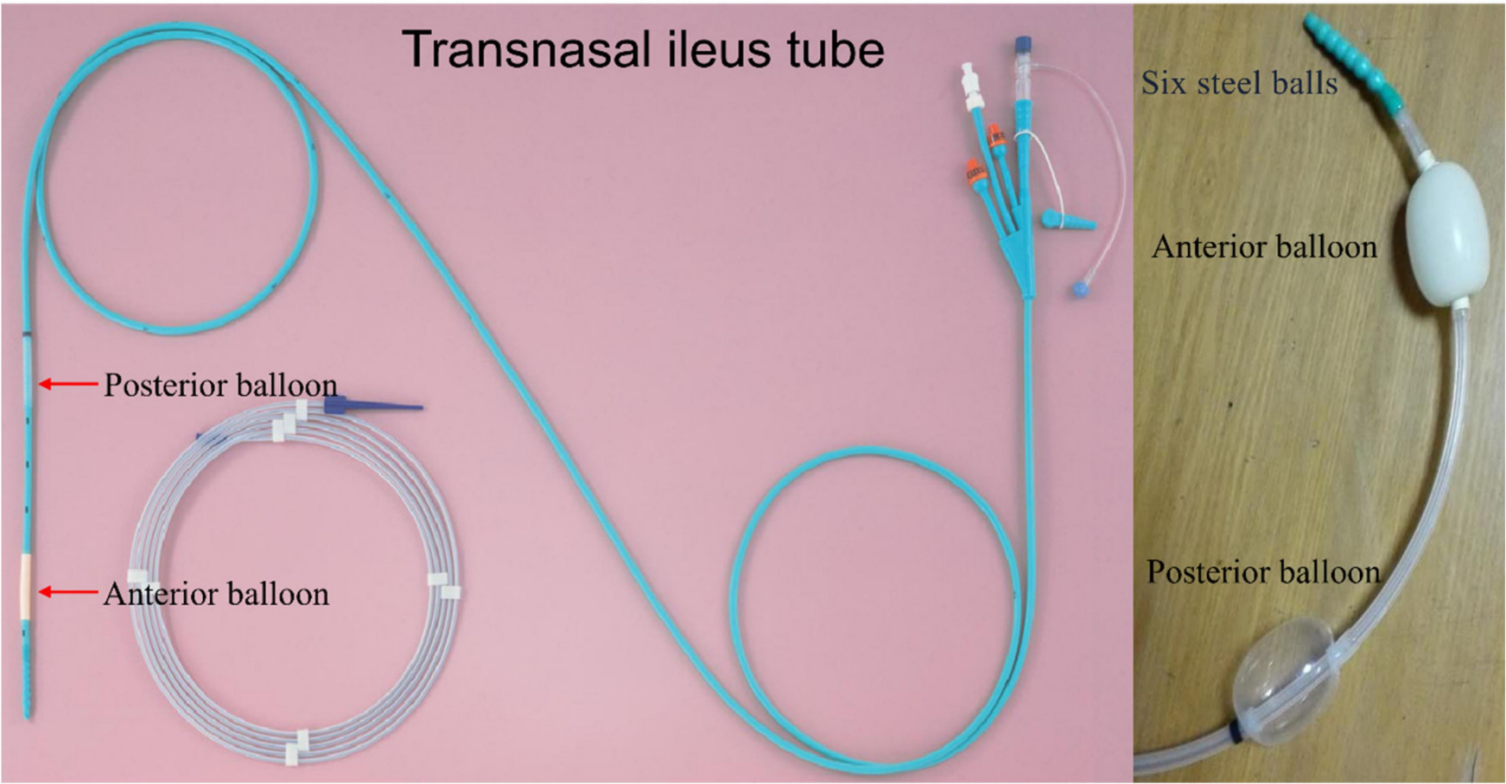
The transnasal ileus tube.

In this study, the CLINY Ileus Tube suite (Create Medic, Tokyo, Japan) was employed (Figure 1). The ileus tube has a length of 300 cm and a diameter of 16 Fr, and it is equipped with three distinct channels: a suction channel, an injection channel with an anti-reflux valve, and a balloon channel. The design incorporates two balloons, referred to as the anterior and posterior balloons. Near the distal end of the tube, there are eight side holes in addition to the terminal hole. Moreover, the tube features a weighted tip consisting of six metal balls to facilitate smoother advancement. The posterior balloon is specifically engineered for application in contrast radiography. The tube permits the injection of water and contrast medium for lavage and imaging purposes. In certain instances, the weighted tip of the tube can directly relieve obstructions. The accompanying guidewire measures 350 cm in length and has a diameter of 1.24 mm (Create Medic).

### Interventional techniques

All procedures were executed under local anesthesia by two interventional radiologists, E.S.L. and J.X.M., with 25 and 18 years of experience, respectively. The interventions were carried out utilizing a single-plane angiography system with fluoroscopic guidance. When required, patients' vital signs, including blood pressure, heart rate, and oxygen saturation (SPO2), were continuously monitored using an electrocardiogram monitor throughout the duration of the procedure.

### Step 1: ileus tube placement

The patient was positioned on the operating table in either a right anterior oblique or supine orientation. Following the administration of topical anesthesia with 2% lidocaine, a tube was inserted through the nasal cavity into the stomach. A 0.035-inch Amplatz super-stiff guidewire was subsequently introduced into the main channel and navigated to the distal duodenum under fluoroscopic guidance. The tube was then advanced over the guidewire into the distal duodenum. Upon withdrawal of the guidewire, the long tube was secured to the patient's cheek. The anterior balloon was inflated with 15 ml of distilled water and propelled forward by bowel peristalsis and its weighted tip. As the anterior balloon advanced, the tube was further progressed. The external end of the tube was connected to a spontaneous negative pressure bag. Intermittent lavage was performed once daily through the long tube, beginning on the second day post-intubation, with careful measurement of the tube's advancement.

### Step 2: pass through the obstructive site

**Figure 2:**
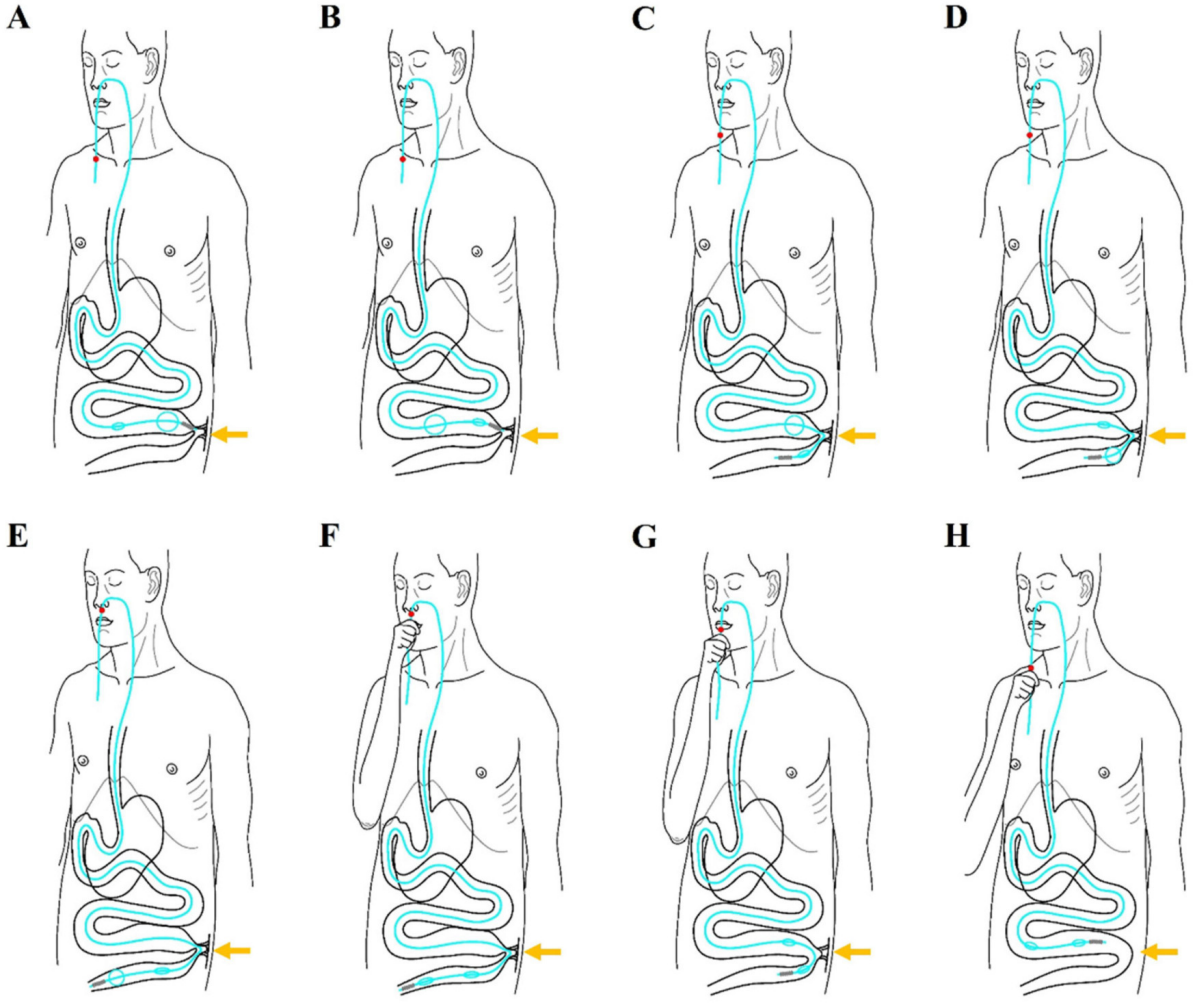
Diagrams illustrates the innovative steps involved in the manual manipulation of a transnasal ileus tube as it traverses an obstructive site, ultimately resolving adhesive obstruction. **(A)** The tip of the ileus tube halts at the proximal obstructive site (indicated by the arrowhead) due to blockage by the obstruction. **(B)** At this stage, the posterior balloon is inflated while the anterior balloon is deflated. **(C)** The inflated posterior balloon facilitates the advancement of the tube's tip through the obstructive site (arrowhead) via bowel peristalsis. **(D)** Upon passing through the obstructive site (arrowhead), the anterior balloon is inflated and the posterior balloon is deflated. **(E)** The anterior balloon then propels the tube's tip further beyond the obstructive site (arrowhead). **(F)** The anterior balloon is deflated once the tip of the ileus tube reaches the colon, with both balloons now deflated. **(G)** The tip of the ileus tube is retracted back through the obstructive site (arrowhead). **(H)** The tube's tip is further retracted to the proximal end of the small bowel, resulting in the loosening of the adhesive obstruction (arrowhead).

Diagrams illustrating the novel procedures for the manual manipulation of the transnasal ileus tube as it traverses the obstructive site are presented in Figure 2 and Supplementary Figure S1. Upon reaching the obstructive site, the ileus tube becomes impeded by the obstruction (Figure 2A). At this juncture, the anterior balloon is deflated while the posterior balloon is inflated (Figure 2B). An angiogram utilizing 76% compound meglumine diatrizoate is conducted to visualize the location and severity of the obstructions. In cases of complete obstruction, it is recommended to maintain the contrast medium within the small bowel for a duration of 12–24 h.

Generally, the combination of the deflated anterior balloon and its weighted tip can be advanced through the obstruction by the inflation of the posterior balloon, aided by bowel peristalsis (Figure 2C). Subsequently, the anterior balloon is inflated, and the posterior balloon is deflated once the anterior balloon has successfully traversed the obstructive site (Figure 2D). The anterior balloon then propels the tip of the tube forward, moving it away from the obstructive site through the action of bowel peristalsis (Figure 2E).

### Step 3: loosening and resolving the adhesive small bowel

Diagrams illustrating the steps for loosening and resolving adhesions in the small bowel are presented in Figure 2 & Supplementary Figure S2. Briefly, the process involves manipulating the ileus tube back and forth to loosen and resolve the adhesions. Once the tip of the tube passes through the obstructive site (Figure 2E), the two balloons are deflated (Figure 2F), and the tube is carefully retracted until the tip is positioned at the proximal small bowel or above the adhesion zones (Figures 2G,H). This procedure can be repeated multiple times until the adhesions in the small bowel are completely loosened and resolved.

Angiographic studies utilizing a 76% meglumine diatrizoate solution, alongside abdominal flat plate radiographs, are performed every 12–24 h post-ileus tube insertion to confirm tube positioning, evaluate the resolution of the obstruction, and inform subsequent clinical decisions. Following the insertion of the long tube, intermittent continuous suction is employed to alleviate intraluminal pressure within the small intestine, while fluid and electrolyte imbalances are addressed. Once the tube's tip reaches the colon, the proximal portion is excised, and the tube is extracted through the anus.

Oral feeding is cautiously reintroduced upon substantial improvement in the clinical manifestations of mechanical intestinal obstruction. Should the patient's condition fail to improve within 72 h post-tube placement, or if there is progression to strangulation, surgical intervention is advised (12)^.^

### Follow-up and postoperative outcome evaluation

Follow-up protocols were implemented at intervals of 1, 3, 6, and 12 months post-removal of the ileus tube, and subsequently on an annual basis. These follow-ups were conducted by one of the two authors and encompassed clinical evaluations, gastroenterography, and abdominal computed tomography (CT) scans. Data pertaining to technical success, initial and final angiographic outcomes, mortality, morbidity, and the ultimate clinical outcome were prospectively collected and analyzed by the authors at the time of patient discharge and at the end of the follow-up period.

Angiographic data were categorized into two classifications: complete resolution of adhesive small bowel obstruction (ASBO), defined by the absence of air-fluid levels and gas in bowel loops, and incomplete resolution, characterized by a reduction of gas and fluid in bowel loops. Clinical follow-ups were classified into four categories: complete recovery from ASBO symptoms and signs, improvement in ASBO symptoms and signs, no change in symptoms, or worsening of ASBO symptoms and signs.

### Statistical analysis

Data are presented as the mean ± standard deviation, and statistical analyses were conducted using SPSS statistical software (version 24.0 for Windows, SPSS Inc., Chicago, IL, USA). A Wilcoxon or Student's *t*-test was used to analyze the difference between the two groups. The statistical *p*-values were two-sided; *p* < 0.05 was considered statistically significant.

## Results

### Patient population

A total of 54 patients (comprising 36 men with a mean age of 53 ± 12 years, ranging from 27–76 years, and 18 women with a mean age of 52 ± 12 years, ranging from 36–82 years) were included in this study. The baseline characteristics of the patients are detailed in Table 1.

**Table 1:** The baseline characteristics of the 54 patients with adhesive small bowel obstruction (ASBO).

Characteristic	Value
Age (years)	53.96 ± 11.06
Female/Male	36/18
complete/incomplete ASBO (no.)	19/35
Duration of ASBO (months)	13.22 ± 5.78 (range, 2–27)
Frequency of ASBO (no.)	2.0 ± 0.87 (range,1–5)
Reasons for ASBO	
Appendectomy	12
Alimentary tract perforation neoplasty	11
Radical correction for Colon cancer	10
Gynecilogical operation	9
Radical correction for Gastric cancer	7
Intestinal necrosis resection	2
Others	3
Duration of the ileus tube placement	24.73 ± 8.65 (range, 9–42 mm)
Number of the obstructions	2.50 ± 0.87 (range, 1–4)
Angiogram follow-ups (months)	15.12 ± 9.29(range, 3–38)
Clinical follow-up (months)	20.23 ± 10.89(range, 6–45)
Final angiogram results (complete occlusion) [no. (%)]	52 (100)
Final clinical results (full recovery) [no. (%)]	52 (100)

### Primary procedural results

**Figure 3:**
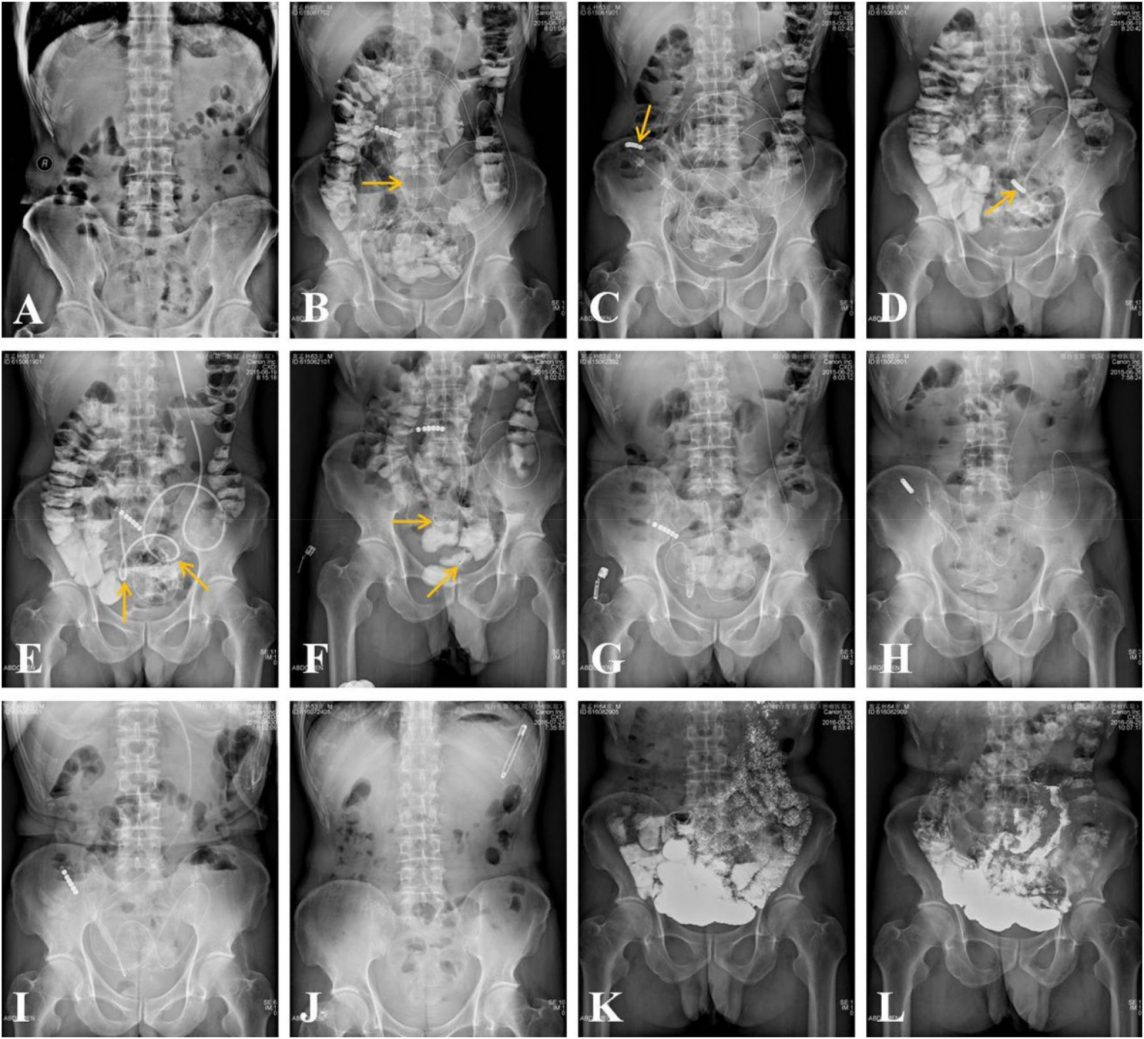
In a 63-year-old male patient, adhesive small bowel obstruction (ASBO) occurred six months following a surgical small bowel resection, which was initially performed to address ASBO secondary to a surgical repair of a stomach perforation that took place 30 years prior. **(A)** An erect abdominal plain radiograph reveals evidence of small bowel obstruction. **(B)** A transnasal ileus tube was inserted into the small bowel under fluoroscopic guidance, with the anterior balloon (indicated by an arrow) inflated with air. **(C)** Three days post-placement, the tip of the ileus tube (indicated by an arrow) reached the right colon. **(D)** The process of retracting the ileus tube to the proximal small bowel took approximately 13 min. **(E)** During the retraction, two obstructive sites (indicated by arrows) were identified. **(F)** Four days post-placement, the retraction of the ileus tube to the proximal small bowel took approximately 5 min, with the two obstructive sites (indicated by arrows) still evident, despite positional changes. **(G-I)** Erect abdominal plain radiographs taken 7, 10, and 15 days post-placement demonstrate a gradual loosening of the adhesive obstruction zones. The morphology of the small bowel transitioned from small to large, from numerous to sparse, and from fixed to flexible. **(J)** The ileus tube was removed 37 days after placement. **(K,L)** Gastroenterography demonstrates unobstruction of small bowel 6 months after the procedures.

**Figure 4:**
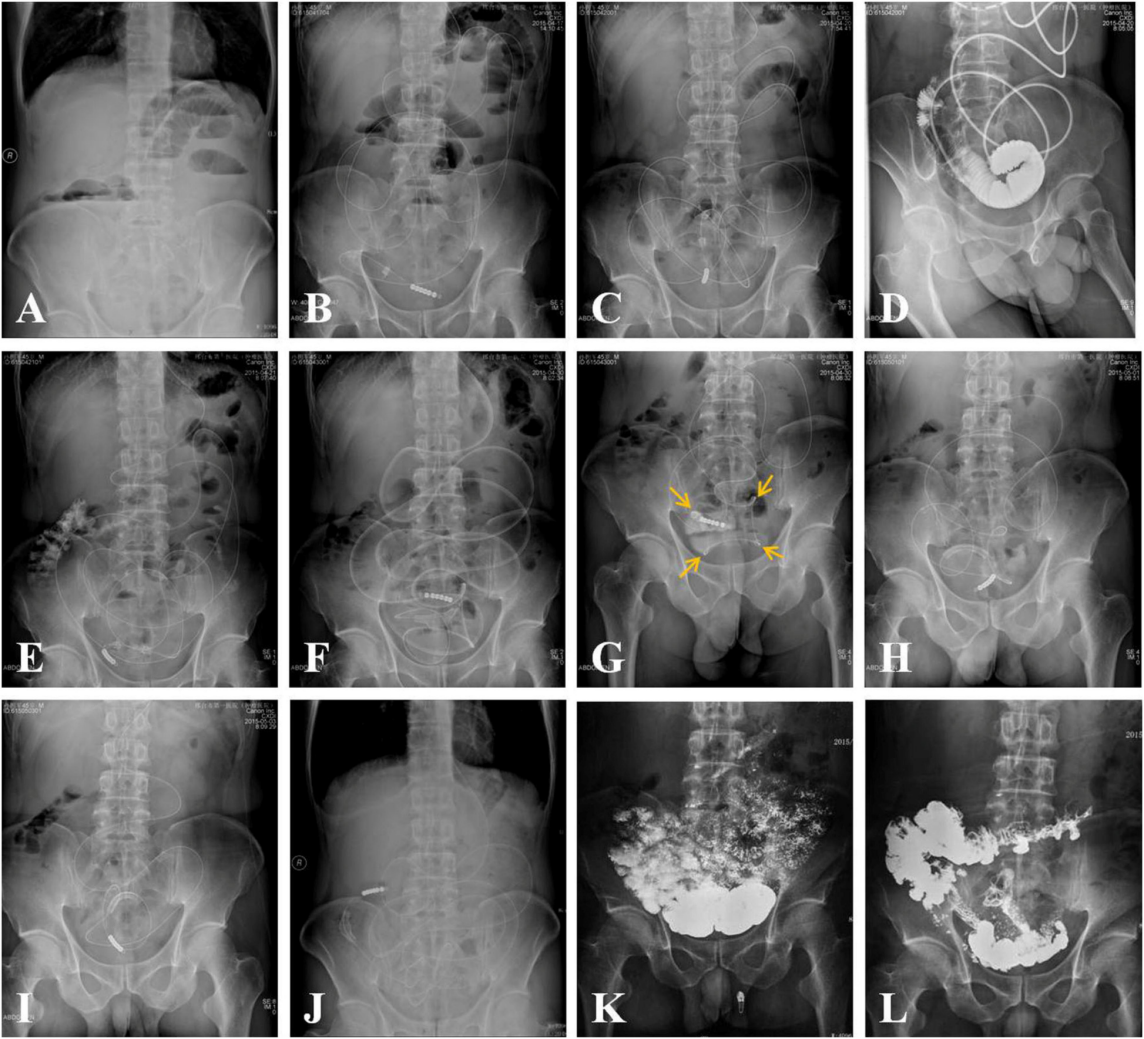
In a 45-year-old male patient, adhesive small bowel obstruction (ASBO) was observed two weeks following radical resection for appendicitis. **(A)** An erect abdominal plain film revealed evidence of small bowel obstruction. **(B)** A transnasal ileus tube was inserted into the small bowel under fluoroscopic guidance. **(C)** Three days post-placement, the advancement of the ileus tube's tip (indicated by an arrow) ceased. **(D)** An angiogram utilizing 76% meglumine diatrizoate demonstrated a complete obstruction of the small bowel. **(E)** After 24 h, the meglumine diatrizoate reached the right colon, indicating a transition from complete to incomplete small bowel obstruction. **(F)** Fourteen days after the ileus tube was placed, its tip reached the distal end of the small bowel. **(G)** Upon retracting the ileus tube, four obstructive sites (indicated by arrows) were identified. **(H-J)** Erect abdominal plain films taken 15, 20, and 24 days post-placement showed a gradual loosening of the adhesive obstructions. As the ileus tube was manipulated back and forth, the curvature of the bowel increased, the tortuosity decreased, and the fixed curvatures diminished. Ultimately, the ileus tube was expelled through the anus. **(K,L)** Gastroenterography demonstrates unobstruction of small bowel 6 months after the procedures.

The comprehensive procedures for the manual manipulation of the transnasal ileus tube to address adhesive small bowel obstruction (ASBO) are depicted in Figures 3, 4. The transnasal ileus tube was successfully placed in all patients without any reported procedure-related complications. Successful navigation and passage of the tube through the obstructions to the colon were accomplished in 51 out of 54 attempts, yielding a technical success rate of 94.4%. In one instance, advancement of the tube was impeded by a true stricture in the small bowel; nonetheless, the patient's clinical symptoms and physical signs of mechanical intestinal obstruction were completely resolved. In two cases, passage through the obstructions was unachievable due to severe obstructions, necessitating surgical small bowel bypass. Postoperatively, complete resolution of clinical symptoms and physical signs of mechanical intestinal obstruction was observed in 52 patients, while partial alleviation was noted in two patients following ileus tube placement. The thirty-day mortality rate was zero.

Excluding the two patients who experienced technique failure, angiographic analysis revealed complete obstruction in 15 patients and incomplete obstructions in 37 patients. A single obstruction was identified in 6 patients, two obstructions in 21 patients (Figure 3), three obstructions in 18 patients, and four obstructions in 7 patients (Figure 4). The mean duration of ileus tube placement was 25 ± 7 days, with a range of 9–42 days. Post-removal angiograms demonstrated that the contrast medium traversed the small bowel without impediment in all 52 patients.

### Follow-up results

At the time of composing this manuscript, follow-up angiograms had been completed for all 52 patients, with a mean follow-up duration of 15 ± 9 months (95% CI: 13, 18 months) and a range of 3–38 months. Angiograms were performed at three and six months for all patients, while 37 patients completed the 12-month follow-up, 15 patients underwent the two-year follow-up, and four patients completed the three-year follow-up. The angiograms obtained at the final follow-up indicated complete resolution of the ABSO in all 52 patients, with no evidence of recurrence.

Clinical follow-up data were available for all patients, with a mean duration of 20 ± 11 months (95% CI: 17, 23 months) and a range of 6–45 months. During this period, one patient succumbed to multiple organ failure due to diffuse metastasis. Importantly, no clinical symptoms or physical signs of mechanical intestinal obstruction were reported by any of the 52 patients, including the patient for whom the ileus tube did not pass to the cecum and the patient who died.

## Discussion

In this study, we introduced an innovative procedure involving the manual manipulation of a transnasal ileus tube for the treatment of severe, multiple, or recurrent adhesive small bowel obstruction (ASBO) and assessed its clinical outcomes. Despite the limited sample size, the transnasal ileus tube successfully navigated obstructions in 94.4% (51 out of 54) of the patients. Complete resolution and full recovery from ASBO were achieved in 100% (52 out of 52) of the patients, with no observed recurrence. The significant improvement in clinical outcomes appears to be primarily attributable to the active adjustment of the two balloons and the back-and-forth manipulation of the tube. These promising results suggest that active manipulation of the transnasal ileus tube is a safe and effective procedure for managing ASBO. Furthermore, a notable finding of this study was the ability to identify the site and number of obstructions, a capability not possible with traditional ileus tubes. The presence of fixed curvatures, indicative of obstructive sites or zones, became apparent when the tip of the tube began to retract during the manipulation process. The site and number of obstructions could be determined based on the location and number of these fixed curvatures.

The ileus tube possesses the capability to aspirate intestinal contents, alleviate bowel wall edema, enhance bowel motility, improve circulation within the affected intestine, rectify intestinal kinking, and prevent bacterial translocation. Unlike traditional ileus tubes documented in the literature, our study introduces notable innovations, particularly through the active manipulation of dual balloons and the bidirectional movement of the tube. The balloons were manually adjusted to navigate obstructions, and the ileus tube was moved back and forth to facilitate the loosening and resolution of adhesive small bowel obstruction (ASBO). This method offers several advantages: 1. successful passage of the transnasal ileus tube through severe, multiple, or recurrent ASBO in the majority of patients, 2. more comprehensive loosening and resolution of ASBO, and 3. a reduced recurrence rate.

In the literature, the traditional ileus tube was characterized by its passive function. It was typically inserted above the distal duodenum, relying on bowel peristalsis to advance the inflated anterior balloon and its weighted tip. This method was generally indicated for cases of incomplete or simple adhesive small bowel obstruction (ASBO), but proved ineffective for complete or multiple ASBOs due to its reliance solely on bowel peristalsis to navigate obstructions. Recent studies on ASBO management using transnasal ileus tubes have demonstrated a resolution rate of approximately 90% among treated patients (9–14). For instance, Guo et al. (15) reported a full recovery in 87.2% of patients following long-tube decompression, without the necessity for surgical intervention, in a cohort of 43 patients with clinical and radiographic evidence of ASBO. Similarly, Chen et al. (16) documented an overall effectiveness rate of 89.6% in a study involving 96 patients treated with an ileus tube, with a recurrence rate of 7% (6 out of 86). Li et al. (17) observed a cure rate of 84% in 25 patients undergoing treatment for recurrent postoperative adhesive ileus with an ileus tube, noting a recurrence rate of 4.8% (1 out of 21) over a follow-up period ranging from 6 months to 2 years.

The passage through the obstruction serves as the foundational premise for the treatment of adhesive small bowel obstructions (ASBOs). The key to effectively treating and preventing the recurrence of ASBOs lies in the repeated advancement and retraction of the ileus tube. Although the balloon may occasionally traverse the obstructions smoothly, this does not necessarily signify that the adhesive zones are completely loosened. In many instances, the adhesive zones are only partially loosened, potentially leading to future recurrence. Therefore, we frequently advance the tip of the ileus tube into the cecum after navigating through the obstructions and subsequently retract the deflated ileus tube to the proximal small bowel. These procedures should be repeated as necessary until the ASBOs are thoroughly loosened and resolved. Throughout the process of advancing and retracting the ileus tube, the small curvatures enlarge, the tortuous sections of the bowel decrease, and the fixed curvatures become less pronounced. Concurrently, the time required to retract the ileus tube from the colon to the proximal small bowel decreases, while the tube traverses the small bowel to the cecum more swiftly than before (Figures 3, 4). The repeated advancement and retraction of the ileus tube not only loosens the adhesive zones but also restores the regular arrangement of the small bowel.

Our findings demonstrated a 100% resolution rate of adhesive small bowel obstruction (ASBO) during follow-up, surpassing the reported range of 90% ASBO resolution in the literature (9–15). This promising outcome suggests that active manipulation of the transnasal ileus tube not only enhances its application but also offers an effective strategy for resolving severe, multiple, or recurrent cases of ASBO. Furthermore, the procedures were not technically challenging; an operator proficient in the transnasal ileus tube technique could successfully execute them.

The advancement of the ileus tube within the small intestine was not consistently successful after its initial placement, often encountering resistance that hindered its progression. Three primary scenarios may account for this impediment: 1. The presence of viscous fecal matter obstructing the tube's forward movement. This issue can often be mitigated by diluting the feces using compound meglumine diatrizoate or saline solutions. 2. Pronounced bowel curvatures, particularly in the jejunum and proximal ileum. This challenge is typically addressed by retracting and repositioning the tube to alleviate the curvature. 3. Obstructions located distal to the tube's tip, which impede its advancement. In such cases, active manipulation of the two balloons is employed to navigate through the obstruction.

When the ileus tube proved ineffective, angiographic procedures employing a 76% solution of meglumine diatrizoate were deemed necessary. Meglumine diatrizoate, a water-soluble contrast agent, is advantageous for both the diagnosis and treatment of adhesive small bowel obstruction (ASBO) (18). In addition to determining the severity and location of the obstruction within the small intestine, meglumine diatrizoate, which remains in the colon for 4–24 h post-administration, can predict the resolution of small bowel obstruction (SBO). Owing to its high osmolarity, this contrast agent draws fluid from intravascular and extracellular spaces into the bowel lumen, thereby reducing bowel edema, diluting fecal matter, promoting proximal bowel distension and peristalsis, and preventing blockage of the suction hole. Simultaneously, the reduction in bowel edema alleviates the severity of the obstruction, facilitating the passage of the ileus tube through the obstructed area, which is considered critical for the treatment of bowel obstruction. However, due to potential risks such as renal failure and anaphylaxis, these agents cannot entirely replace gastrointestinal decompression via an ileus tube (19).

Potential complications, such as intestinal mucosal damage and intestinal rupture, may occur during these procedures; however, they were not observed in this study. Frequent retraction of the ileus tube can exacerbate intestinal mucosal damage; thus, it is generally recommended that this procedure be performed only once daily to facilitate mucosal healing. Additionally, forceful retraction of the ileus tube may lead to intestinal rupture. Patients often perform the retraction themselves, and the procedure should be halted if abdominal pain arises or if increased resistance is encountered during retraction, to prevent bowel rupture. Consequently, it is imperative to consistently remind patients to execute the procedure gently and avoid vigorous movements to mitigate the risk of potential complications.

This study acknowledges several limitations: it was not structured as a comparative analysis, the sample size was limited, and a control group was absent. The primary aim was to elucidate the techniques and outcomes associated with innovative approaches for managing adhesive small bowel obstruction (ASBO) within our clinical practice. Moreover, the procedures utilized to address ASBO entailed considerable radiation exposure due to repeated plain abdominal radiographs and angiograms, although each individual dose was low. Additionally, the complexity of these procedures resulted in prolonged outpatient examination durations. In instances involving true strictures or severe obstructions, the advancement of the ileus tube through the obstructions was challenging.

In conclusion, the manual manipulation of the transnasal ileus tube is non-surgical and therefore non-traumatic but nonetheless an effective method of internally lysing the adhesions with their double balloon technique and pulling the tube back and forth. While the initial results are promising, extended follow-up and larger clinical trials are essential to further substantiate these findings.

## Data Availability

The raw data supporting the conclusions of this article will be made available by the authors, without undue reservation.
