# Supplementary material for: A novel approach of manual manipulation of the transnasal ileus tube for severe or recurrent benign adhesive small bowel obstruction
Source: Front Surg. 2025 Sep 15;12:1601111. doi: 10.3389/fsurg.2025.1601111 (PMC12477208; doi:10.3389/fsurg.2025.1601111)
Supplement: Supplementary file 1 [file Table1.docx]

**
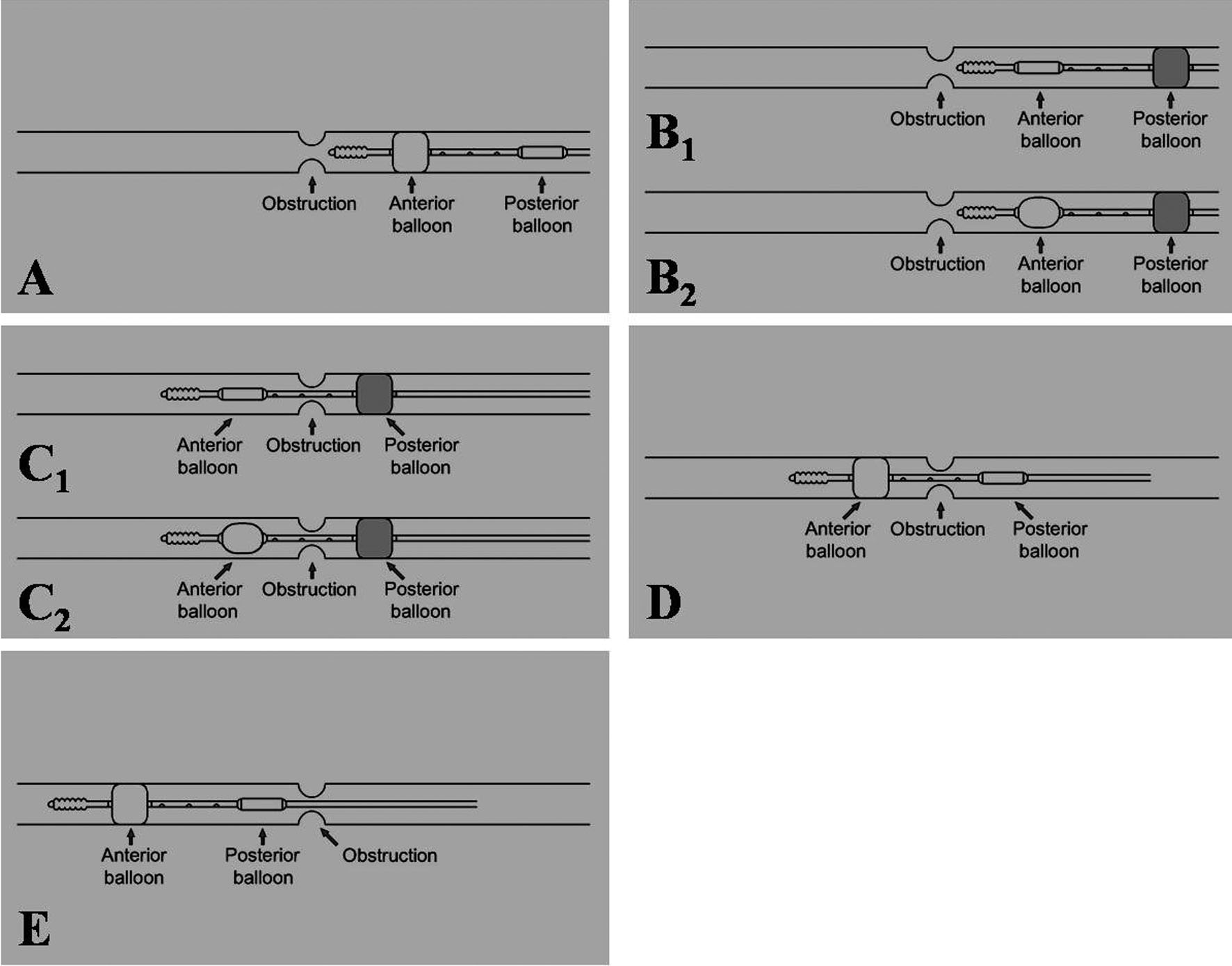
**

**Fig. S1.** Diagrams show the steps of passing the ileus tube through the obstructive site. **(A)** The tip of the ileus tube stops forward at the proximal obstructive site due to blockage by the obstructions. **(B)** Inflates the posterior balloon and deflates the anterior balloon at this time. **(C)** The posterior balloon propels the tip of the tube pass through the obstructive site by bowel peristalsis. **(D)** Inflates the anterior balloon and deflates the posterior balloon when passing through the obstructive site. (**E**) The anterior balloon propels the tip of the tube forward by bowel peristalsis until arrives at ileocecal junction or the right colon. The steps of A→B1→C1→D→E was used for passing through the obstructive site without too much difficulty, and the steps of A→B2→C2→D→E was used for passing through the obstructive site with difficulty.


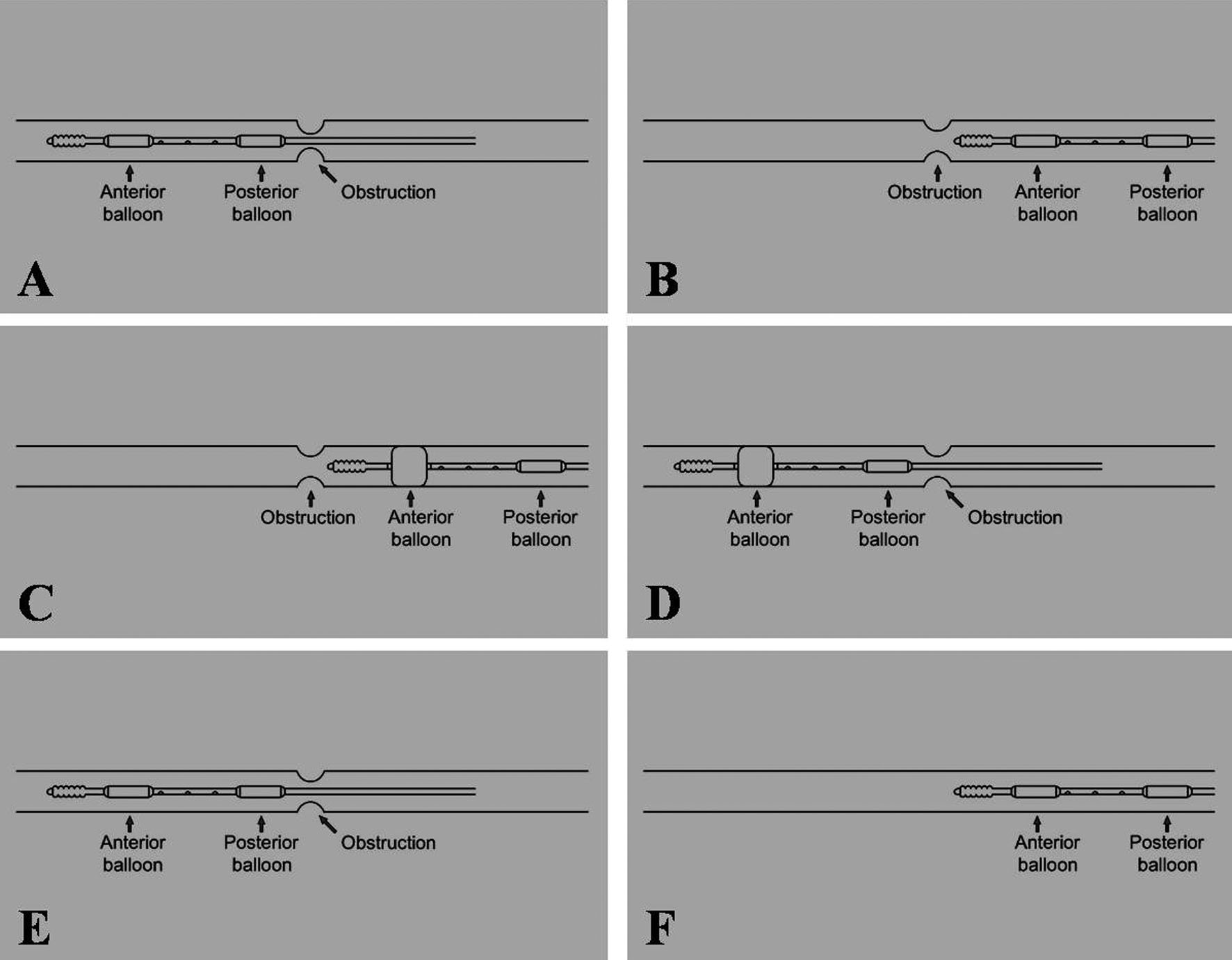


**Fig. S2.** Diagrams show the steps of loosening the adhesive obstructive by draw back the ileus tube repeatedly. **(A)** Deflates the anterior balloon when the tip of the ileus tube arrives at the colon. Note the two balloons are deflated. **(B)** Draw back the tip of the ileus tube to the proximal end of the small bowel. Note the adhesive obstruction is somewhat loosened. **(C)** Inflates the anterior balloon when the tip of the ileus tube arrives at the proximal end of the small bowel. **(D)** The anterior balloon propels the tip of the tube forward by bowel peristalsis until arrives at ileocecal junction or the right colon. **(E)** Deflates the anterior balloon when the tip of the ileus tube arrives at the colon. **(F)** Draw back the tip of the ileus tube to the proximal end of the small bowel again. Note the adhesive obstruction is completely loosened with draw back the ileus tube repeatedly.
